# Synthesis of X-Zeolite from Waste Basalt Powder and its Influencing Factors and Synthesis Mechanism

**DOI:** 10.3390/ma12233895

**Published:** 2019-11-26

**Authors:** Guojun Ke, Haichen Shen, Pengfei Yang

**Affiliations:** 1 School of Civil Engineering, University of South China, Hengyang 421001, Chinashenhaichenjz@163.com (H.S.); 2 Key Laboratory of Special High Performance Concrete, University of South China, Hengyang 421001, China; 3 School of Chemistry and Chemical Engineering, University of South China, Hengyang 421001, China

**Keywords:** basalt, synthesis, zeolites, mechanism

## Abstract

Traditional hydrothermal method (TH) and alkali fusion-assisted hydrothermal method (AFH) were evaluated for the preparation of zeolites from waste basalt powder by using NaOH as the activation reagent in this study. The synthesized products were characterized by BET, XRD, FTIR and SEM. The effects of acid treatment, alkali/basalt ratio, calcination temperature and crystallization temperature on the synthesis process were studied. The results showed that AFH successfully synthesized zeolite X with higher crystallinity and no zeolite was formed by TH. The specific surface area of synthetic zeolite X was 486.46 m^2^·g^−1^, which was much larger than that of original basalt powder (12.12 m^2^·g^−1^). Acid treatment and calcination temperature had no effect on zeolite types, but acid treatment improved the yield and quality of zeolite. Alkali/basalt ratio and crystallization temperature not only affected the crystallinity of synthesized zeolites but also affected its type. The optimum synthesis condition of zeolite X are as follows: acid treatment of 5 wt% HCl solution, NaOH/basalt ratio of 1:1, a calcination temperature of 650 °C and crystallization temperature of 120 °C. The work shows that basalt can be used as a raw material to prepare zeolite.

## 1. Introduction

Zeolites, three-dimensional tetrahedral hydrated aluminosilicate minerals with meso and microporous structures, are widely used as catalysts in separation and refinery industries [1,2,3], feed additives in animal husbandry [4,5], adsorbents in wastewater treatment for the removal of heavy metal cations, anions and dyes [6,7,8]. However, the cost of industrial synthetic zeolites from chemical sources is pretty high, which limits its application to a large extent. Therefore, a lot of attentions have been paid on searching for cheap raw materials for zeolites preparation in recent years. Thirty years ago, Barth-Wirsching and Ulrike [9] achieved the zeolite conversion in the laboratory by simulating the formation of natural zeolites, laying a solid foundation for further study.

So far, various geomaterials and industrial wastes have been used as starting materials for zeolites preparation, such as: Natural kaolin [10], coal gangue [11], coal fly ash [12], bentonite [13], shale rock [14], paper sludge ash [15], bagasse fly ash [16], halloysite [17], waste porcelain [18], lithium slag [19] and so on. With the exploration of raw materials, the synthesis methods of zeolites have been continuously developed. The common methods are traditional hydrothermal method [19], alkali fusion method [20], alkali fusion-assisted hydrothermal method [21], two-step method [22], and sonochemical method [23]. Moreover, in terms of the zeolite synthesis mechanism, there are three relatively developed types, namely solid-phase transition mechanism, liquid-phase transition mechanism and solid-liquid phase transition mechanism.

Coal fly ash (CFA) and other materials are chosen as the starting materials for zeolites synthesis given the high content of Si or/and Al, and given the high content of reactive phases, such as amorphous aluminosilicate glasses. In terms of the standard features, basalt may serve as another ideal raw material. It is the similarity in the chemical composition of basalt and CFA that leads to the testing of basalt based zeolites growth. As well, a large amount of basalt reserves in China make basalt an inexpensive material. Furthermore, the synthesis of zeolites from basalt may enhance its potential value and extend its serviceable scope and reduce the cost of traditional industrial synthetic zeolites. For now, there are few works in literature concerning the synthesis of zeolites from basalt.

The present study centers on the synthesis of zeolites from wasted basalt powder by using TH and AFH, the main objectives being: (a) To obtain an effective synthesis method; (b) to illuminate the activation conditions for maximum synthesis efficiency; (c) to analyze the effects of different synthesis conditions; (d) to reveal the synthesis mechanism of basalt based zeolites.

## 2. Materials and Methods 

### 2.1. Materials

Water, used in the whole experiment, is deionized. Sodium hydroxide (NaOH) and hydrochloric acid (HCl) mentioned below are analytically pure. For CFA, NaOH is more useful to stimulate its activity than KOH and Na_2_CO_3_, hence NaOH is used as the activation reagent in this study. Besides, in order to mix with basalt powder easily, NaOH adopted is granular.

Waste basalt powder was gathered in batches from a basalt mine located in Hunan Province, China. All the collected basalt was mixed well together and ground into fine powders with particle sizes of less than 50 µm (300 mesh sieve).

### 2.2. Synthesis Process

#### 2.2.1. Pretreatment

To study the transition process and mechanism in an even better fashion, some pretreatments of raw basalt powder are designed as follows.

Sample A: Raw basalt powder (particle size ≤50 µm), without treatment. Sample B: Take some sample A into a crucible, annealed in a muffle furnace at 650 °C with a temperature increase rate of 11.5 °C·min^−1^ for 3 h. Mark the annealed solid as sample B. Sample C: Wash some sample A with prepared 5 wt% HCl solution in a sealed beaker at room temperature, followed by vigorous magnetic agitation with a rate of 350 r·min^−1^ for 4 h, in which the liquid/solid ratio is 3:1 (30 mL/10g). After that, separate the solid by centrifugation and wash it with deionzied water in excess. Then dry the washed solid in an electrothermal drying oven at 80 °C for 10 h. Mark the dried product as sample C. Sample D: Treat some sample C with the same method and procedure described in sample B. Then we get sample D. Sample E: Mix 10 g of sample C and 10 g of NaOH uniformly, and treat the mixture with the same method and procedure described in sample B. Grind the annealed lump into powders, namely sample E.

#### 2.2.2. Traditional Hydrothermal Method

This method comprises two major stages: aging and crystallization. First, 10 g of raw material (sample A, B, D) was mixed with 50 mL of 5 M NaOH solution in a beaker. Then the beaker, sealed with preservative film, was set in a water bath for aging at 35 °C with sustained magnetic stirring with a rate of 350 r·min^−1^ for 10 h. Second, transfer the aged mixture into a closed steel vessel lined with PTFE, crystallizing at 120 °C for 12 h. The solid was recovered, washed and dried in the oven at 80 °C.

#### 2.2.3. Alkali Fusion-Assisted Hydrothermal Method

First, 10g of starting material (sample A, sample C), mixed homogeneously with a certain amount of NaOH pellets (5 g, 10 g, 15 g, 20 g) in a crucible, was calcined for alkali fusion in the muffle furnace at a certain temperature (550–750 °C) for 3 h. Then, grind the alkalized lump into powder with a mortar. Blend the powder with 50 mL deionized water for maturing at constant shaking in the 35 °C water bath for 10 h. After that, transfer the matured mixture into a closed steel vessel lined with PTFE for crystallization at a certain temperature (25 °C, 90–150 °C) for 12 h. Last, rinse the crystallographic product with deionized water for a few repetitions. During washing, separate the supernatant precipitate from the lower precipitate. Then dry the washed supernatant precipitate.

### 2.3. Characterization

The bulk chemical compositions of raw basalt sample were determined by X-Ray Fluorescence (XRF, Model: Axios PW4400, Holland). The mineralogical composition of starting materials, intermediate product and synthetic products were analyzed by X-ray diffraction (XRD, Model: Bruker D8, Germany) with diffraction angle (2*θ*) ranging from 5° to 90°. The minerals of samples were identified by matching actual spacing of an unknown mineral. Surface morphology analysis of basalt and synthetic products was performed by scanning electron microscope (SEM, Model: JSM-7500F, Japan). The FTIR spectrum was achieved by means of a FTIR spectrometer (FTIR, Model: Nicolet-460, USA) ranging from 400 cm^−1^ to 4000 cm^−1^. In addition, N_2_ adsorption-desorption was undertaken at 77 K by a volumetric absorption analyzer (BET, Model: ASAP 2020, USA) and the surface area was measured by the BET method.

## 3. Results and Discussion

### 3.1. Properties of Raw Basalt Powder

The bulk chemical composition of raw basalt powder was determined by XRF and given in Table 1. As shown in Table 1, oxides of Si and Al account for about 47.89% and 18.17%, respectively, which act as the fundamental sources of Si and Al needed for zeolites formation. In addition to Al and Si, Fe is the third major element and has an oxide content of 14.67%.

**Figure 1:**
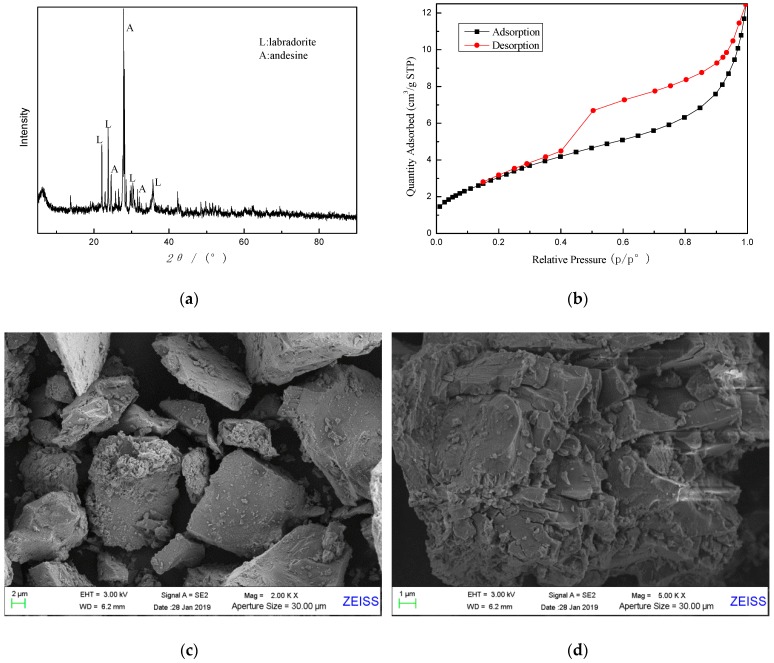
Characterization patterns of raw basalt powder (sample A). (**a**) X-ray diffractogram; (**b**) N_2_ adsorption-desorption isotherm; (**c**) and (**d**) SEM images.

As can be seen from the X-ray diffractogram in Figure 1a, basalt sample is an amorphous phase based on an aluminosilicate glass with minor amounts of crystalline phase substances, mainly labradorite (JCPDS NO. 43-1368) and andesine (JCPDS NO.79-1149). Furthermore, N_2_ adsorption-desorption isotherm of sample A is given in Figure 1b, indicating that sample A has a dense structure with few pores and channels. The specific surface area of raw basalt powder was measured by BET to be about 12.12 m^2^·g^−1^, as shown in Table 2. In addition, some pore-structure parameters are also given in Table 2.

The surface morphology of basalt was characterized by SEM, as shown in Figure 1c,d. As can be seen, the basalt particles are irregular clumps with dense microstructures. Most particles have a relatively smooth surface, and there are no apparent micropores and through holes on the surface, which is consistent with the result from N_2_ adsorption-desorption isotherm.

### 3.2. Comparison of the Two Methods

#### 3.2.1. Traditional Hydrothermal Method

**Figure 2:**
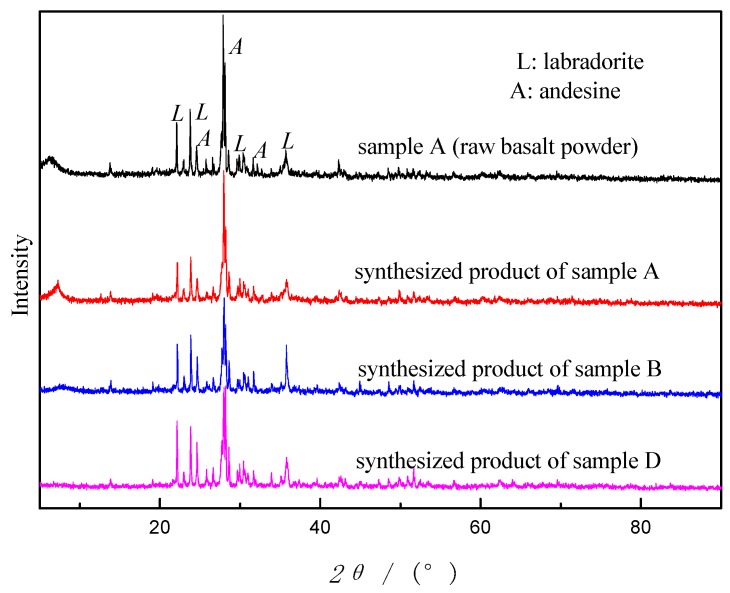
X-ray diffractograms of sample A and synthesized products of sample A, B and D by using TH under the condition: NaOH concentration of 5 M, liquid/solid ratio of 5:1, aging time of 10 h, crystallization temperature of 120 °C, crystallization time of 12 h.

Sample A, B and D were adopted as starting materials by using this method. The results showed that synthesized products of sample A, B and D didn’t show any changes in appearance, still in powder form with the same color as before. All the synthetic products were analyzed by XRD and the diffractograms are shown in Figure 2. As can be seen from Figure 2, as the reaction went on, the peak intensity of the crystal phases in sample A gradually declined but did not disappear. Noteworthily, no new peaks were showing. The crystalline phases of all the synthetic products were the same as sample A, without zeolite crystals. No zeolites were synthesized. The results turned out that TH is not an effective way to prepare zeolites from basalt powder.

Nevertheless, this method was successfully performed to form zeolites from CFA by Doyle A M et al. [24]. That it did not work well on basalt is possibly attributed to the quite different surface morphologies of CFA and basalt. The CFA particles are spherical, but basalt particles are mainly irregular lumps with dense microstructures, which means that basalt has a lower contact area for reaction with OH^−^ than CFA. On the other hand, the compact inner structure of basalt blocks the spreading pathway for OH^−^, which may weaken the dissolution of Si oxides and Al oxides.

In addition, as can be seen from Figure 2, after the reaction the peak intensity of the crystalline phases decreases, which signifies that some labradorite and andesine were digested during synthesis process. From the differences of the descending extent, we may conclude that calcination and acid treatment activate reactivity of basalt to some extent, concurring with the conclusion on CFA [24].

#### 3.2.2. Alkali Fusion-Assisted Hydrothermal Method

**Figure 3:**
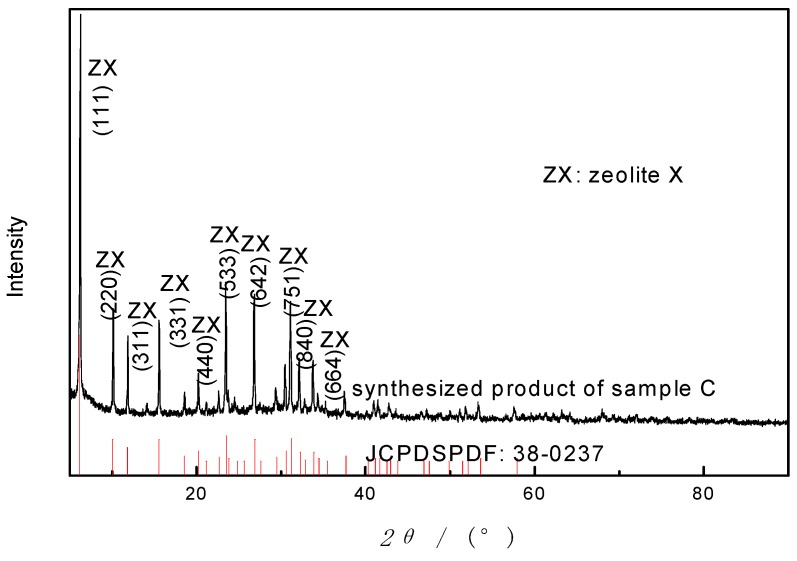
X-ray diffractogram of synthetic product of sample C by AFH under the condition: NaOH/basalt ratio of 1:1, calcination temperature of 650 °C, aging time of 10 h, crystallization temperature of 120 °C, crystallization time of 12 h.

In this method, a mixture of NaOH and starting material (sample A and C) was heated in high temperature (550–750 °C) for alkali fusion before hydrothermal treatment. The XRD pattern of synthesized product is shown in Figure 3. As can be seen in Figure 3, following the synthesis course, the peaks of crystalline minerals in basalt decline and disappear; new crystals occur and grow. The characteristic diffraction peaks of the sample were stable without obvious impurity peak, and the peak width is narrow and sharp, indicating that the product has high purity, complete structure and single crystal phase. In addition, there are seven peaks with strong intensity, at 2*θ* of 6.3°, 10.1°, 11.9°, 15.6°, 23.5°, 26.8° and 31.1°, respectively. The diffraction peaks are identical with Na_2_Al_2_Si_2.5_O_9·_6.2H_2_O (zeolite X, JCPDS PDF 38-0237) [25]. Zeolite X was successfully synthesized by this method. The experiment result illustrated that AFH is an effective way to synthesize zeolites from basalt powder and alkali fusion is more effective to dissolve the aluminosilicate minerals than hydrothermal treatment, which concurs with the synthesis of kaolin zeolites [26].

Essentially, the fusion of the NaOH and basalt powder mixture facilitates the formation of highly active Na-silicates and Na-aluminates, which are readily dissolvable in aqueous solution and enhance zeolite formation significantly. The higher concentration of aluminosilicates, Na-silicates and Na-aluminates in the reaction system can precipitate zeolites more easily.

#### 3.2.3. Characterization of Zeolite X

Besides XRD pattern above, to study the physicochemical properties of synthetic zeolite X under optimum condition, further characterizations were carried out by SEM, BET and FTIR.

**Figure 4:**
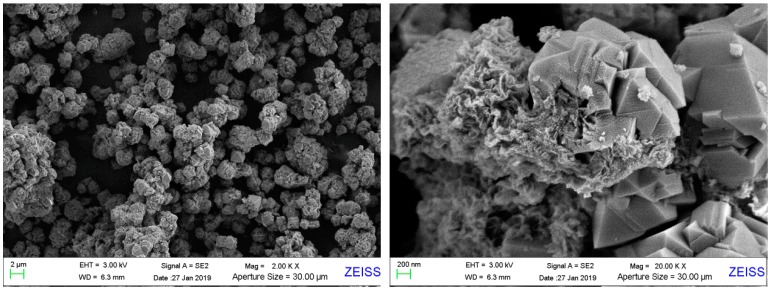
SEM images of synthesized product of sample C by using AFH under the condition: NaOH/basalt ratio of 1:1, calcination temperature of 650 °C, aging time of 10 h, crystallization temperature of 120 °C, crystallization time of 12 h.

The surface morphological structure of the synthesized product was determined by SEM, as shown in Figure 4. As clearly shown in Figure 4, the crystallites occur and grow with different sizes based on the rotted basalt particle. Lots of ZX crystallites cluster together surrounding the basalt particles, like blossoming flowers. There are only a few single crystals. That is possible because the crystalsare fed and grow in a relatively calm environment without stirring. The crystallites, are mainly octahedral-shaped, accompanied by some littery crystals in atactic shape, which is in accord with the morphology of faujasite (zeolite X is a typical type of faujasite). The morphological analysis of SEM images is in accord with the result of XRD above.

**Figure 5:**
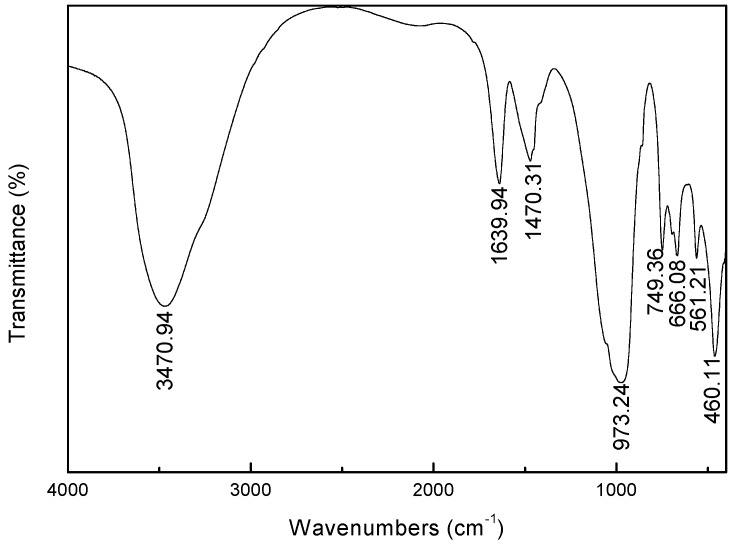
FTIR spectrum of zeolite X synthesized by AFH under the condition: NaOH/basalt ratio of 1:1, calcination temperature of 650 °C, aging time of 10 h, crystallization temperature of 120 °C, crystallization time of 12 h.

FTIR spectrum of synthesized zeolite X is shown in Figure 5. The single strong adsorption band at 3470.94 cm^−1^ is attributed to OH^−^ stretching of water molecules in the zeolite caves. The band at 1639.94 cm^−1^ ascribed to H_2_O deformation mode because of incomplete dehydration of the synthetic zeolite, indicating that there is free water present in the zeolite structure. Moreover, the typical bands, attributed to asymmetric stretch mode (973.24 cm^−1^), symmetric stretch mode (666.08 cm^−1^), double six-member rings (D6R, 561.21 cm^−1^) and a bending mode of the T-O bond (460.11 cm^−1^), are observed (where T is Al or Si). The spectra data is consent with lithium slag based zeolite X [27].

**Figure 6:**
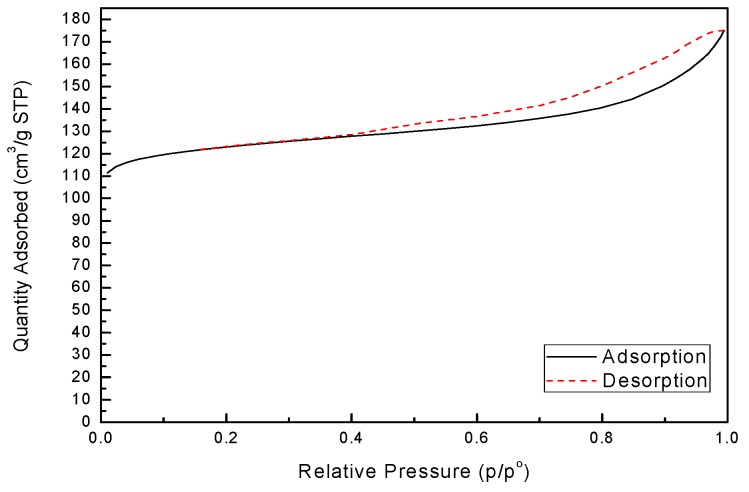
N_2_ adsorption-desorption isotherm of synthesized zeolite X.

N_2_ adsorption-desorption isotherm of zeolite X, as shown in Figure 6, was undertaken to calculate the BET surface area, pore volume and pore size and the result is given in Table 3. From Figure 6, zeolite X is structured with micropores, quite different from basalt shown in Figure 1b. The specific surface area of synthetic zeolite X was measured by BET to be 486.46 m^2^·g^−1^, lower than pure zeolite X of 720 m^2^·g^−1^ [28], but much larger than basalt sample of 12.12 m^2^·g^−1^.

### 3.3. Effects of Different Synthesis Conditions

#### 3.3.1. Acid Treatment

As shown in Table 1, the basalt sample is a Fe-rich type, with Fe oxides accounting for about 14.67%. According to the related literature [29], iron oxides in the CFA is known to be undesirable for zeolites formation. So acid pretreatment was designed to remove Fe_2_O_3_ in the raw basalt powder. Table 4 shows three main components of basalt powder obtained after acid treatment by 5 wt% HCl solution. It indicates a reduction of about 6.5% of iron components and increases of about 11.76% and 6.18% in silicon and aluminum compounds, respectively. After acid washing, the sample color turns light grey from dark grey, which may be explained by the reduction of Fe oxides.

**Figure 7:**
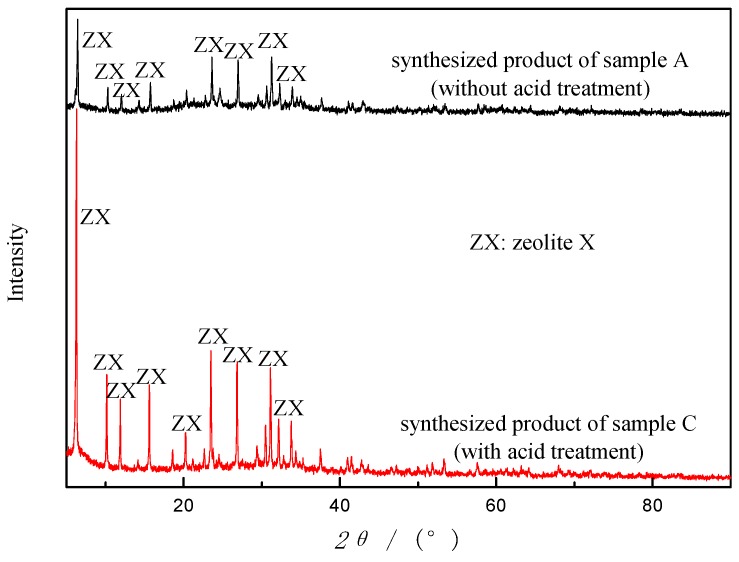
X-ray diffractograms of synthetic products from sample A and sample C by AFH under the same condition.

Sample A and sample C were both adopted under the same synthesis condition to study the effects of acid treatment. From Figure 7, zeolites were generated from both sample A and C, with the same single crystalline phase (ZX). However, from the intensity of the peaks, the zeolite product of sample C has a much higher degree of crystallinity than that of sample A. The removal of Fe by acid washing may explain the improvement of crystallinity. In addition, after acid treatment, the yield increases (2.375 g vs. 1.420 g from 10 g of starting material), and the whiteness and fineness of ZX enhances, which is consistent with the prior study of CFA zeolites [29]. Accordingly, acid treatment enhances the yield and quality of synthesized zeolites but does not affect zeolite types.

Acid treatment may also leach a certain amount of Al_2_O_3_, which is located in the outer surface of basalt particles. Thus, it relatively improves the SiO_2_ content of the reaction system and the Si/Al ratio may increase to some extent. However, in this study, the synthetic crystalline phase didn’t show any changes, which may be explained in terms of the process used. In addition, previous works show that faujasite could be formed with Si/Al ratio ranging from 2 to 5 [30].

Since acid treatment is in favor of zeolite formation, sample C is used in the following experiments.

#### 3.3.2. Alkali/Basalt Ratio

**Figure 8:**
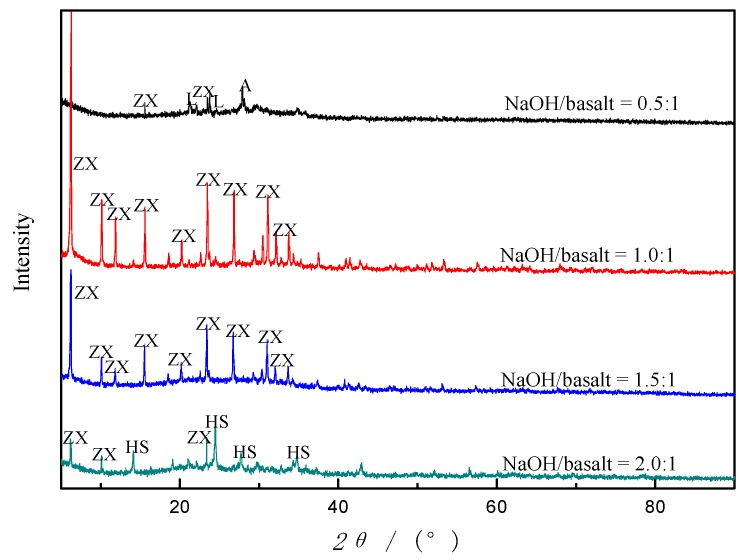
Diffractograms of products synthesized by using AFH with different NaOH/basalt ratios (Designation: L: labradorite; A: anorthite; ZX: zeolite X; HS: hydroxylsodalite).

Alkali fusion is a general approach to decompose materials rich in silicon or/and aluminum. NaOH present in the reaction system acts as an activator for the formation of soluble aluminate and silicate salts, which are the sources of Si and Al for zeolite synthesis. A series of experiments were undertaken to analyze the effects of NaOH/basalt ratio on zeolite formation and the products were evaluated by XRD as shown in Figure 8. The results showed that there was only a little zeolite (zeolite X) yielded when the alkali/basalt ratio is lower than 0.5:1. However, what is noteworthy is that most of the prior crystalline phases in basalt were melted into amorphous silicates and aluminates compounds, as shown in Figure 8. AFH is more lethal to the crystalline phases and amorphous component in basalt than TH, which verifies the conclusion in comparison of the two methods. When the ratio is up to 1:1, the ZX crystal sprang up like mushrooms with a high degree of crystallinity, with prior crystal phase disappearing. With the NaOH/basalt ratio increasing, the intensity of the ZX reflections decreases without other crystal phases showing up. Following the disappearance of ZX peak, zeolite hydroxylsodalite (HS, JCPDS NO. 41-0009) occurred when the ratio is around 2.0:1. Furthermore, when the ratio is above 2:1, the annealed lump becomes very tough, as well as the annealed product of a pasty mixture of basalt and NaOH, which is very hard to be ground into a powder and not used for further study.

From the disappearance of labradorite and anorthite peaks, it is deduced that both labradorite and anorthite in the basalt powder have reacted with NaOH. Alkali fusion is very effective in extracting silicon and aluminum species in basalt powder, by which labradorite and andesine in basalt were melted in 3 h. However, the fusion of minerals in basalt lies a foundation for zeolites synthesis. Alkalinity and concentration of Na^+^ play an important role in the synthesis course of zeolites. Low alkalinity (alkali/basalt ratio of 0.5:1) cannot provide the appropriate condition for rearranging Si^4+^, Al^3+^ and Na^+^ into zeolites crystal. Na^+^ is known as the stabilizer of the sub-building units of zeolite frameworks [30]. Therefore, NaOH/basalt ratio plays a crucial role in the formation of the zeolites, which affects not only the degree of zeolite crystallinity but also the synthesized zeolite types.

#### 3.3.3. Calcination Temperature

**Figure 9:**
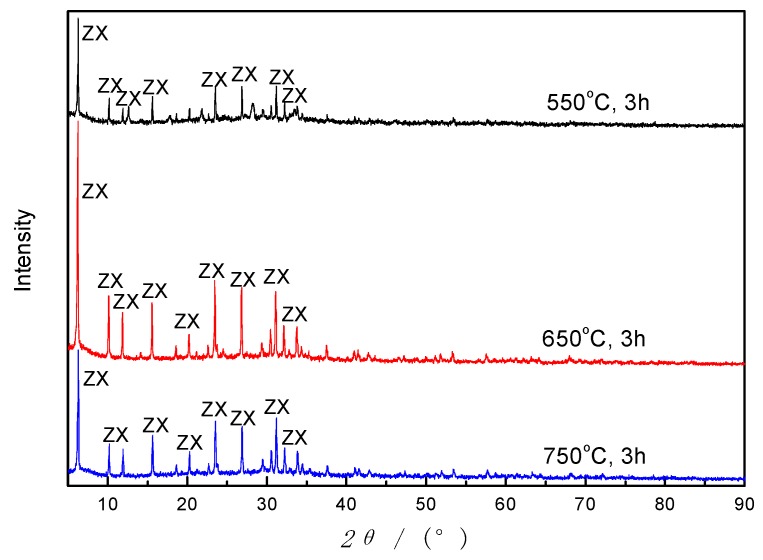
Diffractograms of products synthesized by using AFH under different calcination temperatures.

Generally, a higher calcination temperature could enhance the amount of Si^4+^ and Al^3+^ extracted from basalt powder, as well as the reaction efficiency. However, a previous study on formation of CFA zeolites indicated that too high calcination temperature may go against zeolite formation [30], because the Si and Al fusion compound may convert into other crystalline phases instead of zeolite crystal. So, three moderate temperature levels are set in this study, and the result is given in Figure 9.

As demonstrated in Figure 9, ZX was formed under different calcination temperatures, but with different degrees of crystallinity. When the temperature is lower than 650 °C, the peak intensity increases with the increase of calcination temperature, and then descends over 650 °C without the crystal phases changing. Furthermore, the yield increases gradually as the calcination temperature increases. Accordingly, in a specific range of temperature (550–750 °C), calcination affects the output of synthesized zeolites and its crystallinity, but not the zeolite types.

#### 3.3.4. Crystallization Temperature

**Figure 10:**
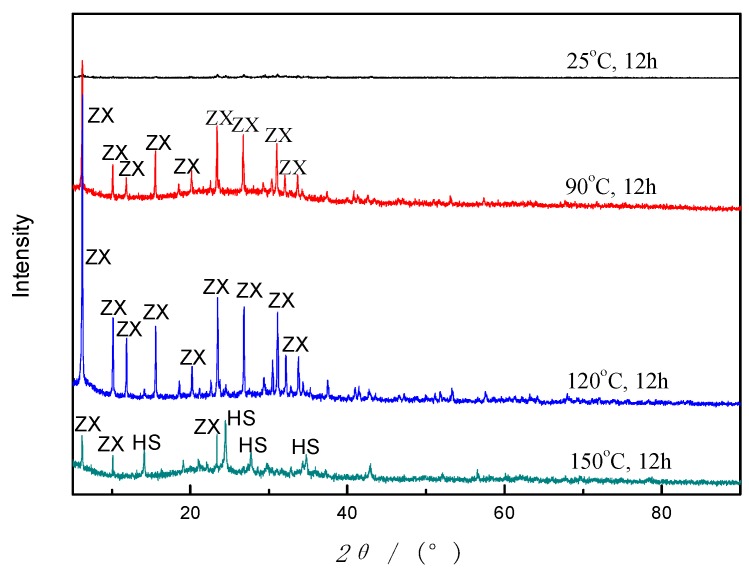
Diffractograms of products synthesized by using AFH under different crystallization temperatures.

Crystallization is the process that builds the crystal framework of zeolites and that occurs faster at higher temperatures. According to the related literature [31], the crystalline phase will transfer to other types when the temperature is over a specific value. Therefore, a series of experiments about crystallization temperatures were carried out, and the products were analyzed by XRD, as given in Figure 10. It is shown that there are no distinct peaks of crystal phases at room temperature (25 °C), which indicates that low temperature is not suitable for zeolites formation. As temperature rises to about 90 °C, the ZX crystals gradually show up and grow rapidly, reaching a plateau at 120 °C. Nevertheless, zeolite HS occurs and replaces zeolite X bit by bit when the temperature is beyond 120 °C. Quite mounts of zeolite HS were formed when the crystallization temperature is up to 150 °C. This behavior is not occasional. Mostly, faujasite type zeolites have a larger specific surface area and pore size than hydroxysodalite. So, it is an unstable phase in the reaction system, tending to convert into other stable types when the condition changes [31]. In addition, the yield increases as the temperature increases. It is inferred that 120 °C is a relatively suitable crystallization temperature for ZX formation.

### 3.4. Synthesis Mechanism

**Figure 11:**
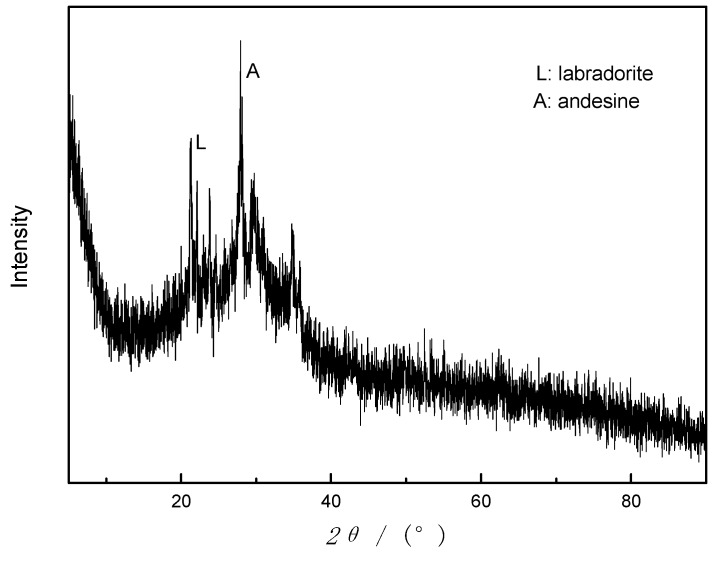
X-ray diffractogram of sample E.

Acid pretreatment removes the undesirable Fe_2_O_3_ located in the surface of the basalt particle, enhancing the relative content of Si_2_O_3_ and Al_2_O_3_ indirectly. In the alkali fusion stage, OH^−^ spreads into the system, surrounding the basalt particles and permeating into the channels. The reactive phase was decomposed gradually, resulting in the concentration of Al^3+^ and Si^4+^ increasing. However, the dissociation course would not last all the time and reach an equilibrium as the alkalinity descends and as the Na-aluminate and Na-silicate salts covered the surface of basalt particle. During this fusion period, no zeolite crystals are occurring, as shown in Figure 11, which is consistent with the liquid phase transition mechanism.

This process can be described as follows:

(1) $$NaOH+xAl_{2}O_{3}\cdot ySiO_{2}\overset{Fusion}{\to }Na_{2}SiO_{3}+Na_{2}AlO_{2}$$

In the aging stage, Na^+^, Al^3+^ and Si^4+^ were dissolved into aqueous solution. Stirring facilitates the release of Al^3+^ and Si^4+^. As we know that Si^4+^ reacts readily with Al^3+^ precursors to generate aluminosilicates compounds. When enough quantity of silicate ions was generated in the reaction system, aluminates and silicates are condensed to form an aluminosilicate gel onto the surface of basalt particles, as depicted in the equation:

(2) $$NaOH(aq)+Na_{2}Al{\left(OH\right)}_{4}(aq)+Na_{2}SiO_{3}(aq)\overset{Aging}{\to }[Na_{x}(AlO_{2}){\left(SiO_{2}\right)}_{2}\cdot NaOH\cdot H_{2}O](gel)$$

In the crystallization stage, crystallization of zeolites takes place through nucleation reaction and crystal growth. In alkaline conditions, aluminum constitutes negatively charged tetrahedral species, a structure coinciding with the zeolite framework. A previous work shows that there exists an incubation period of 3h during which the nucleation occurs [31]. Thereafter, zeolite X crystal grows rapidly for a few hours. However, ZX is an unstable phase in comparison to hydroxysodalite. Once beyond some critical point, the equilibrium will be interrupted, resulting in a collapse in the ZX framework and being rearranged into HS crystal. The period may be demonstrated with the reaction equation, as below:

(3) $$\left[Na_{x}(AlO_{2}){\left(SiO_{2}\right)}_{2}\cdot NaOH\cdot H_{2}O](gel)\overset{Crystallization}{\to }Na_{p}[{\left(AlO2\right)}_{p}{\left(SiO2\right)}_{q}\cdot hH_{2}O\right]$$

## 4. Conclusions

(a) Compared to TH, AFH is apracticalapproach for zeolite preparation from basalt. Zeolite X can be synthesized with high purity and crystallinity by using AFH.

(b) Acid treatment, alkali/basalt ratio, calcination temperature and crystallization temperature play a significant role in the conversion from basalt to zeolites. The acid treatment enhances the yield and quality of synthetic zeolites; calcination temperature (550–750°C) affects the degree of crystallinity, but not the synthesized zeolite types. NaOH/basalt ratio and crystallization temperature affect not only the degree of zeolite crystallinity but also the synthesized zeolite types.

(c) The quality and type of the synthesized zeolites could vary significantly, depending on the formation conditions and parameters. The optimum synthesis condition of zeolite X was acid treatment of 5wt% HCl solution, NaOH/basalt ratio of 1:1, calcination temperature of 650 °C and crystallization temperature of 120 °C.

## Figures and Tables

**Table 1:** Chemical composition of raw basalt powder.

Components	SiO_2_	Al_2_O_3_	Fe_2_O_3_	CaO	MgO	Na_2_O	K_2_O	SO_3_	Ignition Loss	Total
wt.%	47.89	18.17	14.67	5.61	4.3	2.10	1.50	0.06	3.18	97.48

**Table 2:** Surface area and pore-structure parameters of raw basalt powder.

Sample	Surface Area (m^2^·g^−1^)	Mircopore Volume (t-Plot) (cm^3^·g^−1^)	Pore Volume (cm^3^·g^−1^)	Pore Size (nm)
Sample A	12.1196	0.002750	0.019297	6.36874

**Table 3:** Surface area and pore-structure parameters of synthesized zeolite X.

Sample	Surface Area (BET) (m^2^·g^−1^)	Mircopore Volume (t-Plot) (cm^3^·g^−1^)	Pore Volume (cm^3^·g^−1^)	Pore Size (nm)
Zeolite X	486.4602	0.171909	0.270844	2.22706

**Table 4:** Main chemical composition of basalt powder after acid treatment (sample C).

Components	SiO_2_	Al_2_O_3_	Fe_2_O_3_
wt%	59.65	24.35	8.23
